# A new addition to the embalmed fauna of ancient Egypt: Güldenstaedt’s White-toothed Shrew, *Crocidura gueldenstaedtii* (Pallas, 1811) (Mammalia: Eulipotyphla: Soricidae)

**DOI:** 10.1371/journal.pone.0249377

**Published:** 2021-04-07

**Authors:** Neal Woodman, Salima Ikram, Joanne Rowland

**Affiliations:** 1 U.S. Geological Survey, Leetown/Patuxent Wildlife Research Center, Laurel, Maryland, United States of America; 2 Department of Vertebrate Zoology, National Museum of Natural History, Smithsonian Institution, Washington, District of Columbia, United States of America; 3 Department of Sociology, Egyptology, and Anthropology, American University in Cairo, New Cairo, Egypt; 4 Department of Ancient Studies, Stellenbosch University, Stellenbosch, South Africa; 5 Department of Archaeology, School of History, Classics, and Archaeology, The University of Edinburgh, Edinburgh, Scotland; Hebrew University, ISRAEL

## Abstract

The Falcon Necropolis at Quesna in the Nile Delta of Egypt is considered to have been founded by the priest Djedhor, the Saviour, of Athribis (Tell Atrib in modern Benha) at the beginning of the Ptolemaic Period. Recent excavations here have revealed abundant avian remains from mummies dedicated to the ancient Egyptian god Horus Khenty-Khety. Among the few mammal remains from the site are five species of shrews (Eulipotyphla: Soricidae), including some that we identified as Güldenstaedt’s White-toothed Shrew, *Crocidura gueldenstaedtii* (Pallas, 1811). Discovery of this species at Quesna increases the number of shrews recovered from ancient Egyptian archaeological sites to eight species. *Crocidura gueldenstaedtii* no longer occurs in the Nile Delta, and its presence in a diverse shrew fauna at Quesna that includes one other extirpated species, *Crocidura fulvastra* (Sundevall, 1843), supports the hypothesis of a moister regional environment 2000–3000 years ago. Inadvertently preserved local faunas, such as that from Quesna, can provide valuable information about ancient environments and subsequent turnover in faunal communities.

## Introduction

Mummification and burial of animals for religious purposes began early in Egyptian history, culminating in the construction of extensive animal necropoleis during the Late (c. 712–332 BC), Ptolemaic (c. 332–30 BC), and Early Roman (30 BC–AD 250) periods [1–5]. Prominent among the embalmed animals are canids, felids, ibises, and raptors whose cumulative mummified remains each number in the millions [6–11]. Among the less numerous mammals deemed worthy of mummification were shrews (Eulipotyphla: Soricidae), which by the time of the late New Kingdom (1550–1069 BC), were associated with the dark (nighttime) aspect of the falcon-headed god Horus, in contrast to that god’s light (daytime) aspect, represented by diurnal raptors [2, 4, 6, 11–15].

Six species of shrews have previously been identified from mummified remains recovered at ancient Egyptian archaeological sites [16]. They include two Egyptian endemics [*Crocidura floweri* Dollman, 1915; *C*. *religiosa* (Geoffroy Saint-Hilaire, 1827)], an extinct species (*C*. *balsamifera* Hutterer, 1994), a species that no longer occurs in Egypt [*C*. *fulvastra* (Sundevall, 1843)], and two wide-ranging species whose distributions include northern Egypt [*C*. *olivieri* (Lesson, 1827); *Suncus etruscus* (Savi, 1822)]. More recently, Rainer Hutterer identified a seventh species, *Crocidura pasha* Dollman, 1915, among the animal mummies in the Passalacqua [17] collection of antiquities from the tomb of Queen Mentuhotep at Dra Abu el-Naga near Thebes (modern Luxor) [18]. A second record of *C*. *pasha* has since been reported [19] from the Spanish Mission’s excavations of tombs associated with two high court officials named Djehuty and Hery [20], also at Dra Abu el-Naga. Like *C*. *fulvastra*, this species no longer inhabits Egypt.

Herein, we report the discovery of an eighth species of embalmed shrew, *Crocidura gueldenstaedtii* (Pallas, 1811), from a collection of remains recovered from the Falcon Necropolis at Quesna in the Nile Delta of Egypt [20]. Discovery of this species at ancient Quesna adds to the evidence for a more diverse Egyptian shrew fauna occupying the Nile Valley in the historical past. It also has biogeographical implications for the species, which currently reaches its southwestern distributional limit in the Sinai Peninsula.

## Materials and methods

### Locality

**Fig 1:**
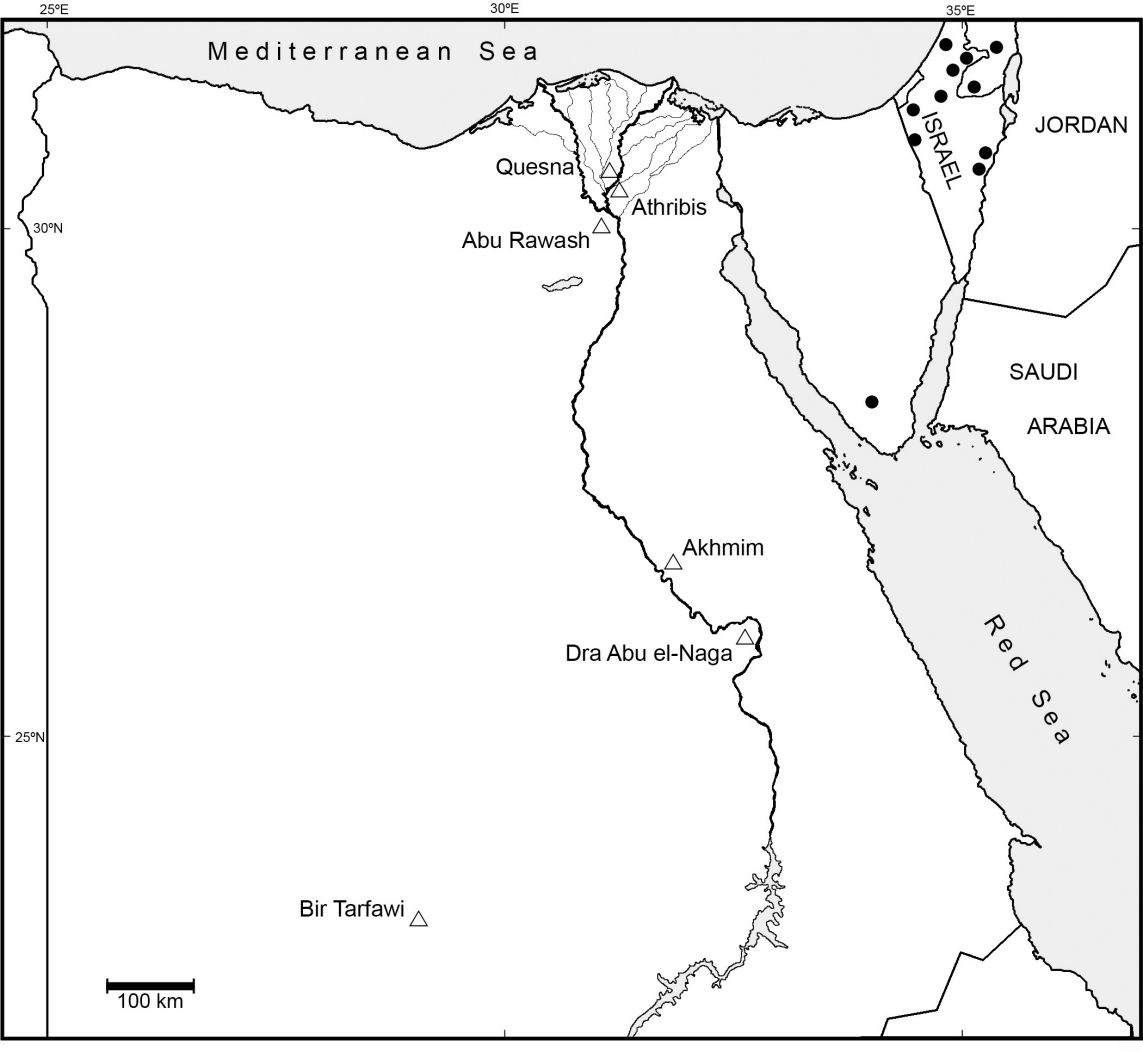
Map of Egypt. Locations of archaeological sites mentioned in the text are shown as open triangles. Localities for modern *C*. *gueldenstaedtii* appear as closed circles and are based on Shpirer et al. [30] and Osborn and Helmy [33].

The archaeological site of Quesna (ca. 30° 31’ 54” N, 31° 10’ 18” E) is located approximately 3.5 km east of the modern town of the same name in Minufiyeh Governorate, Egypt (Fig 1). Texts on mud seal impressions from recent excavations in the Falcon Necropolis link Quesna with Athribis [11], which is 7 km south and is the site from which Djedhor, the priest of the cult of the raptor god Horus, is known [11, 21–23]. All small mammal remains studied were taken from among concentrations of disarticulated bones excavated from the Falcon Necropolis. This collapsed and buried mud-brick structure (hypogeum) was probably founded during the time of Philip Arrhideaus (332–323 BC) at the very start of the Ptolemaic Period (332–30 BC) and later extended. It was used as a necropolis for animal mummies dedicated to the god Horus [11, 24]. The majority (98%) of remains from the Falcon Necropolis are from avians, principally from Falconiformes [11]. The remainder are primarily from small mammals, most notably shrews.

### Identification

Shrew remains from the Falcon Necropolis were identified, measured, and photographed. Measurements of shrew crania and dentaries were recorded to the nearest 0.1 mm and follow Carraway [25] and Hutterer and Kock [26]. These include length of lower toothrow, P_4_ to M_3_ (p4-m3); height of coronoid process of the dentary (HCP); maxillary breadth (MXB); and palatal length (PL) (S1 Dataset). Measurements from remains of smaller shrews (i.e., smaller than *Crocidura olivieri*) were compared to those of modern specimens of *C*. *floweri*, *C*. *fulvastra*, *C*. *religiosa*, *C*. *gueldenstaedtii*, and *C*. *whitakeri* de Winton, 1887 (S1 Appendix), housed in the following collections: Field Museum of Natural History, Chicago, USA (FMNH); Natural History Museum, London, UK (NHMUK); University of Michigan Museum of Zoology, Ann Arbor, USA (UMMZ); National Museum of Natural History, Washington, USA (USNM); and Yale Peabody Museum of Natural History, New Haven, USA (YPM). Cranial measurements of modern *C*. *pasha* are values reported by Hutterer and Kock [27].

The taxonomic history of what is now called *C*. *gueldenstaedtii* is complex. We follow Burgin et al. [28] in recognizing *C*. *gueldenstaedtii* as a species in the *C*. *suaveolens* group [29–31], rather than as a subspecies of *C*. *suaveolens* (Pallas, 1811). As currently constituted, *C*. *gueldenstaedtii* includes several subspecies (*C*. *g*. *cypria* Bate, 1903; *C*. *g*. *iculisma* Mottaz, 1908; *C*. *g*. *mimula* Miller, 1901) from western Europe and the Mediterranean island of Cyprus that potentially represent distinct species. We focus our discussion on *C*. *g*. *gueldenstaedtii*, whose geographic distribution extends from western Iran, Azerbaijan, Georgia, and eastern Turkey, southwest along the eastern shore of the Mediterranean Sea to Israel and the Sinai Peninsula [28, 29, 31, 32]. This taxon is the modern member of the *C*. *suaveolens* group that is geographically closest to Quesna. Specimens of *C*. *gueldenstaedtii* from the Sinai were previously identified as *Crocidura suaveolens portali* Thomas, 1920 [33–35].

## Results

**Fig 2:**
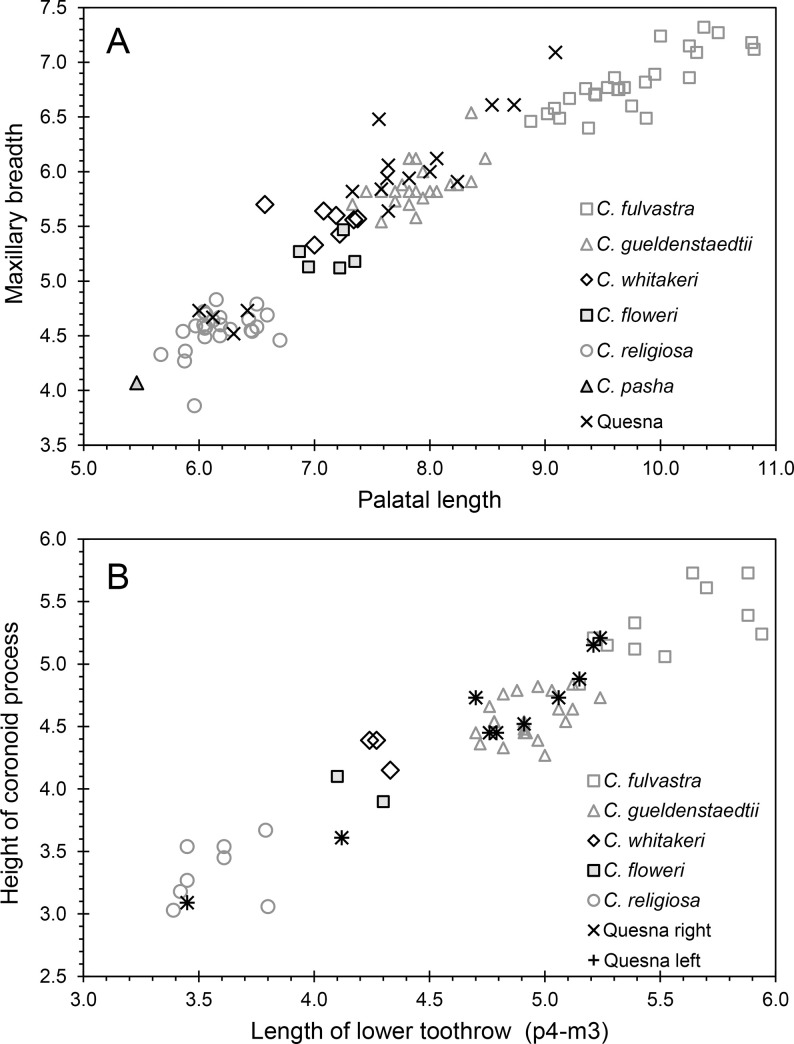
Comparisons of smaller shrew specimens at Quesna with those of modern specimens. A, plot of cranial variables; B, plot of dentary variables.

**Fig 3:**
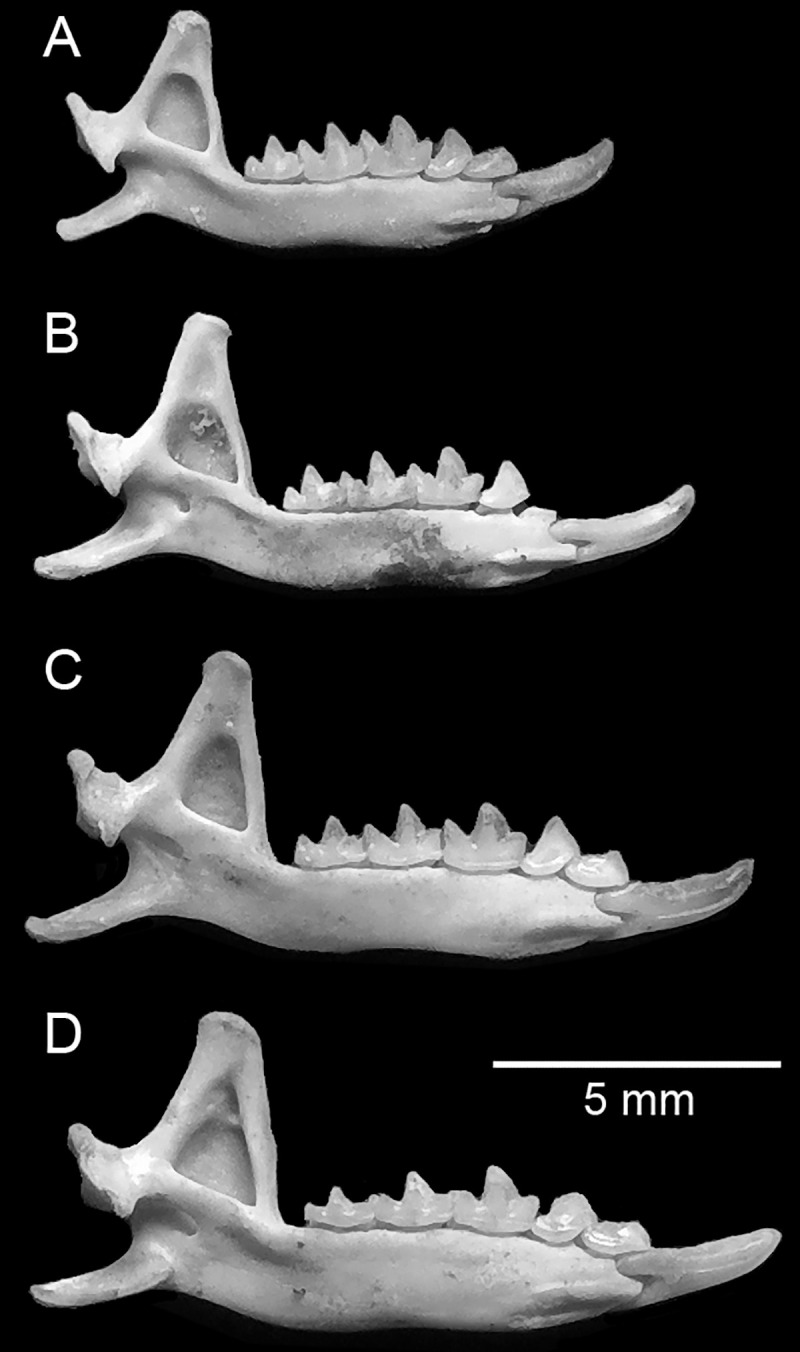
Lingual view of left dentaries of preserved shrews at Quesna. A, *Crocidura religiosa*; B, *C*. *floweri*; C, *C*. *gueldenstaedtii*; D, *C*. *fulvastra*.

**Fig 4:**
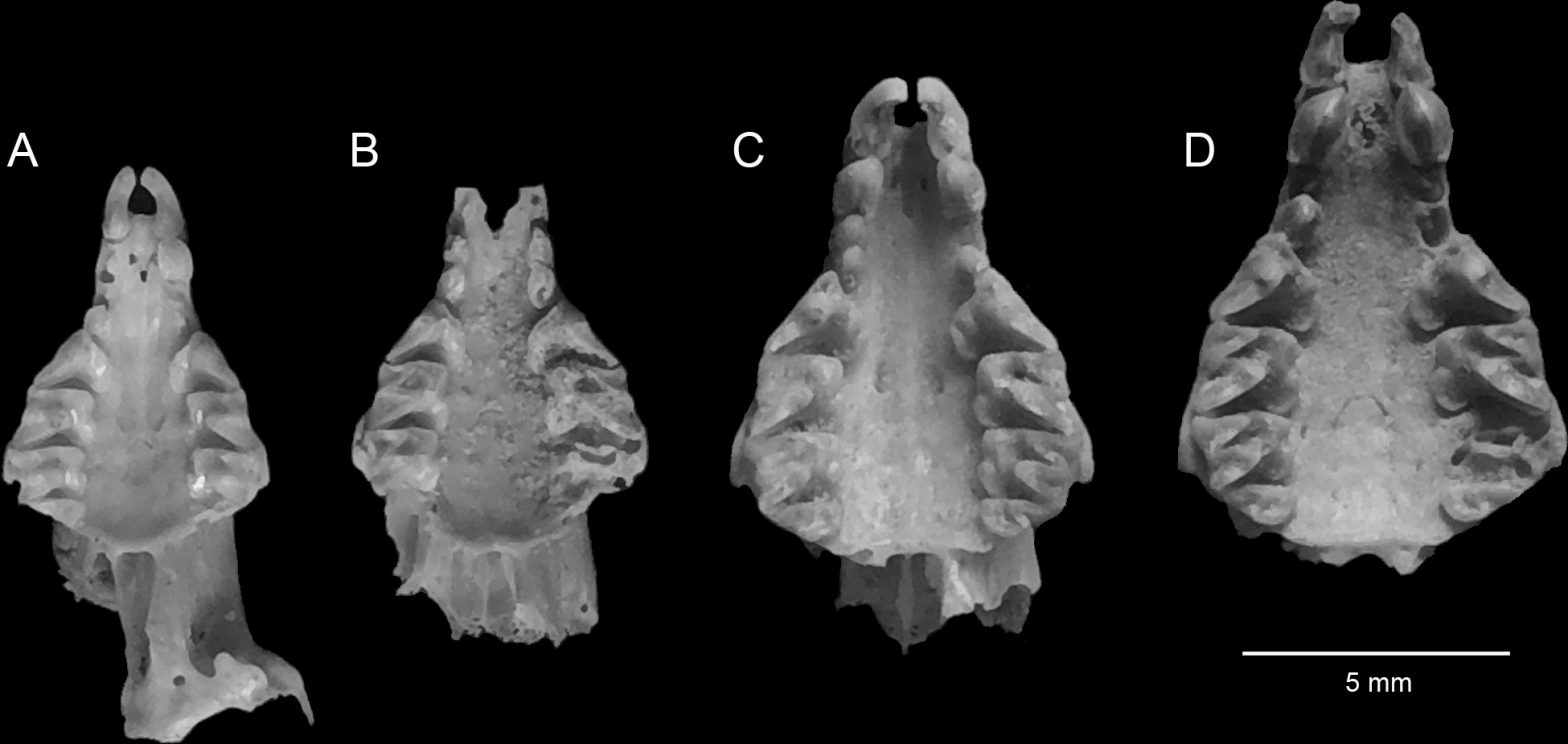
Palatal view of crania of preserved shrews at Quesna. A, *Crocidura religiosa*; B, *C*. *floweri*; C, *C*. *gueldenstaedtii*; D, *C*. *fulvastra*.

We recovered 80 identifiable elements from a minimum of 33 individuals representing four species of smaller soricids. These included 3 *Crocidura floweri*, 9 *C*. *fulvastra*, 7 *C*. *religiosa*, and 14 *C*. *gueldenstaedtii* (Table 1). Specimens identified as *C*. *gueldenstaedtii* average larger in most measurements of the cranium and dentary, and they have more robust dentition than modern *C*. *pasha*, *C*. *religiosa*, *C*. *floweri*, and *C*. *whitakeri*, but they are smaller and have less robust dentition than *C*. *fulvastra* (Figs 2–4; Table 1).

**Table 1:** Selected cranial and dentary measurements.

Species	PL	MXB	HCP	p4-m3
*Crocidura pasha* ^a^	5.5	4.2	3.0	⸻
	5.4–5.6	3.8–4.3	2.8–3.2	
	(*n* = 5)	(*n* = 18)	(*n* = 18)	
*C*. *religiosa*	6.2 ± 0.3	4.6 ± 0.1	3.3 ± 0.2	3.5 ± 0.2
	5.7–6.7	4.3–4.8	3.0–3.7	3.4–3.8
	(*n* = 22)	(*n* = 22)	(*n* = 5)	(*n* = 5)
*C*. *floweri*	7.1 ± 0.2	5.2 ± 0.1	4.0, 4.0	4.2, 4.2
	6.9–7.4	5.1–5.5		
	(*n* = 5)	(*n* = 5)	(*n* = 2)	(*n* = 2)
*C*. *whitakeri*	7.1 ± 0.3	5.6 ± 0.3	4.3 ± 0.1	4.3 ± 0.05
	6.6–7.4	5.3–6.2	4.2–4.4	4.2–4.3
	(*n* = 7)	(*n* = 8)	(*n* = 3)	(*n* = 3)
*C*. *gueldenstaedtii*	7.8 ± 0.3	6.0 ± 0.2	4.6 ± 0.1	4.8 ± 0.1
(Quesna)	7.3–8.2	5.6–6.5	4.3–4.8	4.7–5.1
	(*n* = 14)	(*n* = 10)	(*n* = 25)	(*n* = 9)
*C*. *gueldenstaedtii*	7.9 ± 0.3	5.9 ± 0.2	4.6 ± 0.2	4.9 ± 0.2
(modern)	7.3–8.5	5.5–6.5	4.3–4.8	4.3–5.2
	(*n* = 23)	(*n* = 23)	(*n* = 23)	(*n* = 23)
*C*. *fulvastra*	9.8 ± 0.5	6.8 ± 0.3	5.3 ± 0.3	5.6 ± 0.3
	8.9–10.8	6.4–7.3	4.8–5.7	5.2–5.9
	(*n* = 27)	(*n* = 27)	(*n* = 11)	(*n* = 12)

Selected cranial and dentary measurements of ancient Egyptian *Crocidura gueldenstaedtii* at Quesna compared with those from six modern species of *Crocidura*. Abbreviations of measurements are given in the Materials and Methods. Statistics are mean ± *SD* and range. Sample sizes are in parentheses.

^a^ Measurement statistics from Hutterer and Kock [27].

## Discussion

We initially expected that the individuals we identify herein as *C*. *gueldenstaedtii* would prove to be either *C*. *floweri* or the slightly larger *C*. *whitakeri*. In fact, remains of *C*. *floweri* were identified from Quesna, but they were less numerous than those of *C*. *gueldenstaedtii*. *Crocidura floweri* currently is restricted to the Nile Delta and the Fayum [36]. In addition to our specimens from Quesna, remains of this species have been reported from ancient Egyptian archaeological sites elsewhere in the Nile Delta, such as Abu Rawash [37, 38], and in the Nile Valley as far south as Akhmim [16, 38]. Prehistoric records of *C*. *floweri* from the South Galala Plateau in northern part of the eastern desert [39] and from Middle Pleistocene lake deposits at Bir Tarfawi in southern Egypt [40] indicate this species once had a much wider distribution in Egypt than at present.

*Crocidura whitakeri* is primarily distributed in coastal regions of Western Sahara, Morocco, Algeria, and Tunisia. Disjunct populations of the species also occur along Mediterranean coastal Egypt west of the Nile Delta near Marsa Matruh and in the northern Sinai along Lake Bardawil. Egyptian *C*. *whitakeri* were formerly identified as *C*. *suaveolens matruhensis* Setzer, 1960 [16, 34, 35]. Although *C*. *whitakeri* has not been identified from any archaeological sites in Egypt, its modern distribution suggests that it would not be unlikely for it to have had a wider distribution that included the Nile Delta in the historical past.

Remains of the somewhat larger *Crocidura fulvastra* also occurred in some abundance at Quesna. The modern distribution of this species is discontinuous in dry savanna and mesic habitats across central Africa from Mali to southern Sudan and western Ethiopia [41]. Although it no longer occurs in Egypt, remains of this species have been recovered from a number of archaeological contexts, including at Dra Abu el Naga [19] and at Akhmim [16, 42].

The intermediate size of *C*. *gueldenstaedtii* between the smaller *C*. *floweri* and the larger *C*. *fulvastra* raises the possibility that archaeological remains of this shrew from other sites have been misidentified as one or the other species. This confusion would be understandable given the complicated taxonomic history of *C*. *gueldenstaedtii*, the limited modern distribution of this species in Egypt, the relative scarcity of modern comparative specimens from Egypt and neighboring regions, and the more general difficulties involved in identifying remains of soricids, particularly when they are incomplete or they are wrapped up in mummy bundles.

Dubey et al. [31] modeled the long-term population dynamics of *C*. *gueldenstaedtii* based on their molecular phylogeny of the *C*. *suaveolens* group. They hypothesized that southern populations of the species had expanded from a bottleneck that may have resulted from global cooling c. 17,300–22,100 yr BP during the last glacial maximum in Europe. Discovery of remains of *C*. *gueldenstaedtii* at Quesna in the Nile Delta indicates that population expansion had reached its peak in the Middle East and northeastern Africa by the Ptolemaic period and that the population there has since contracted eastward from the Nile Delta. *Crocidura gueldenstaedtii* and the other species of shrews in the Falcon Necropolis represent populations that inhabited the delta during a moist climatic phase c. 3000–2000 yr BP within a longer-term regional trend of aridification and desertification beginning c. 5500–5000 yr BP [43–47] that has made the region less hospitable for soricids [19] and other animals [48, 49].

Particularly in their animal mummy deposits, the ancient Egyptians unknowingly preserved a partial record of the local small mammal faunas that inhabited the area at that time. Documenting mammalian and other faunal assemblages permits researchers to examine how both individual species and the communities to which they belonged have responded to past climate change in the region [19]. It is also potentially important for understanding how current species and communities may react to future climatic change predicted by current climatic models [50]. Shrews are particularly good for monitoring local and regional conditions, and we know that among the greater diversity of species present at ancient Egyptian sites, one is now extinct [16] and two are extirpated from the region [18, 19]. The comprehensive analysis necessary to understand regional faunal turnover in shrew, mammal, and other communities, however, requires a greater number of ancient sites with accurately and verifiably identified species.

## Supporting information

S1 Dataset Measurements from shrew remains. (XLS)

S1 Appendix Modern specimens examined. (DOCX)
