# Supplementary material for: A new addition to the embalmed fauna of ancient Egypt: Güldenstaedt’s White-toothed Shrew, Crocidura gueldenstaedtii (Pallas, 1811) (Mammalia: Eulipotyphla: Soricidae)
Source: PLoS One. 2021 Apr 7;16(4):e0249377. doi: 10.1371/journal.pone.0249377 (PMC8026016; doi:10.1371/journal.pone.0249377)
Supplement: S1 Appendix — (DOCX) [file pone.0249377.s002.docx]

**S1 Appendix: Modern Specimens Examined**

***Crocidura floweri*** (*n* = 7).⸻Egypt: Beheira Governate: Wadi el Natrun, 5.5 km N Lake el Zugun (UMMZ 165633, 165633). Giza Governate: Giza (NHMUK 10.6.18.2, 10.6.18.3⸻holotype; 10.6.18.6; 10.6.18.7); Kafr el Sheikh Governate: 1 km S Baltim Beach (FM 106469).

***Crocidura fulvastra*** (*n* = 28).⸻South Sudan: Al-Istiwa'Iyah Ash-Sharqiyah, Kapoeta (USNM 317888); Upper Nile Province: Paloich, Niayok (FM 93701, 93702, 93704, 93706, 93709; USNM 325928, 325929, 325930, 325931, 325932); Paloich, 1.6 km N Niayok (FM 93712); Paloich, 19 km N Niayok (FM 93711; USNM 325933); Paloich, Tir, Paloi (FM 93843; USNM 325934); Paloich, 1.6 km NE Tir (FM 93844); Paloich, 3.2 km N Tir (FM 98973); Paloich, 8 km NE Tir (FM 93846; USNM 325935); Paloich, Tir, Toich, Thaak (FM 93848; USNM 325936, 325937); Paloich, Tir, Toich, 3.2 km S Thaak (FM 93851); Paloich, Kum Kum Forest (FM 96213). Sudan: Southern Kordofan Province: Buk (FM 29008). Eastern Equatoria Province: Kapoeta (FM 85173). Kassala Province: Aroma Madart, on River Gash, 21 km SSE of Kassala (USNM 325927).

***Crocidura gueldenstaedtii*** (*n* = 24): Iran: Azarbaijan-E Gharbi Province: 11 km N Rezaiyeh, (USNM 354509); Azarbaijan-E Sharqi Province: 5 km SE Meyaneh (USNM 354503, 354504, 354505, 354514, 354517, 354519). Lorestan Province: 50 km SW Borujerd (USNM 350117). Kermanshah Province: 42 km W Kermanshah (USNM 354501, 354502, 354507, 354508, 354511). Turkey: Icel Province: Tarsus (USNM 327226, 327227, 327228, 327229); 8 km S Namrun, on Cehennem Dere (USNM 327230, 327231, 327232, 327233, 327234, 536423, 536424).

***Crocidura religiosa*** (*n* = 28).⸻Crania: Egypt: Cairo (NHMUK 1904.8.2.4, 1904.8.2.5, 1904.8.2.6, 1904.8.2.7, 1904.8.2.8); East Bank of Nile, University Farm, Assuit (NHMUK 1976.547). Giza Governate: Giza (NHMUK 1910.6.18.4, 1910.6.18.5); Imbaba, Abu Rawash (FMNH 84653, 89580, 89985, 95891, 98169, 100739, 106443; NHMUK 1916.8.16.2, 1916.8.16.3; USNM 311766, 341933; YPM 5840); Imbaba, Minshat el Bakkari (FMNH, 84753); Imbaba, Kafr Hakim (USNM 311767); Imbaba, Nahya (FMNH, 84567; USNM 311768). Luxor Governate: Luxor Necropolis of Karnak (UMMZ 165634, 165635, 165636). No locality (FMNH, 108383).

***Crocidura whitakeri*** (*n* = 7).⸻Algeria, Ain Sefra (NHMUK 13.8.6.11, 13.8.6.12). Egypt, Matruh, 4.8 km W Marsa Matruh (FMNH 91184). Morocco: Sierzet, about halfway between Morocco city and Mogador (NHMUK 98.7.4.5⸻holotype); Safi, 5 km E Essaouira (USNM 482019, 48220, 485838).
